# Stillbirth in the African Great Lakes region: A pooled analysis of Demographic and Health Surveys

**DOI:** 10.1371/journal.pone.0202603

**Published:** 2018-08-29

**Authors:** Blessing Jaka Akombi, Pramesh Raj Ghimire, Kingsley Emwinyore Agho, Andre Masumbuko Renzaho

**Affiliations:** 1 School of Science and Health, Western Sydney University, Penrith, New South Wales, Australia; 2 School of Social Sciences and Psychology, Western Sydney University, Penrith, New South Wales, Australia; The University of Warwick, UNITED KINGDOM

## Abstract

**Background:**

The aim of this study was to estimate the rate and predisposing factors associated with stillbirth in the African Great Lakes region (Burundi, Congo Democratic Republic, Kenya, Rwanda, Tanzania and Uganda).

**Methods and findings:**

Cross-sectional data from the most recent Demographic and Health Surveys (DHS) of countries in the African Great Lakes region were used in this study. DHS from Congo Democratic Republic was not included in the analyses because data was not collected for stillbirth in the country survey. A pooled sample of 57046 pregnancies of 7+ months’ duration and 1002 stillbirths were included in the final analysis. The analyses were restricted to stillbirths reported in the 5 years preceding the surveys. Stillbirth was defined as foetal death in the third trimester (≥ 28 weeks’ gestation). Multilevel logistic regression analyses that adjusted for cluster and survey weights were used to determine the factors associated with stillbirth in the Africa Great Lakes region. Health service variables and maternal medical condition variables were not included in the analysis because DHS do not collect data on these variables for pregnancies that did not result in a live birth. Burundi had the highest stillbirth rate per 1000 births [23% (95% CI: 20, 25)] within the region. Factors associated with stillbirth across the region were: no schooling [1.85 (95%Cl: 1.44, 2.38)] and primary education [1.64 (1.32, 2.05)], advanced maternal age [2.39 (95% CI: 1.59, 3.59)], smoking [1.99 (95% CI: 1.19, 3.32)] and drinking water from unimproved sources [1.18 (95% CI: 1.01, 1.37)].

**Conclusion:**

To achieve Every Newborn Action Plan (ENAP) stillbirth target of 12 per 1000 births or less by 2030, policy interventions to prevent stillbirth should focus on promoting community-based socio-educational programmes which encourages a healthy lifestyle especially among uneducated women in the advanced age spectrum.

## Introduction

Stillbirth is a public health as well as a development problem in low and middle-income regions. According to World Health Organization (WHO), stillbirth refers to third trimester foetal death (≥ 28 weeks’ gestation) [1]. Stillbirth could either be antepartum or intrapartum. Antepartum stillbirths, also known as macerated or intra-uterine stillbirths, occur if the baby dies in the womb before the onset of labor, usually more than 12 hours prior to delivery. Intrapartum stillbirths, also referred to as fresh stillbirths, occur if the baby dies after the onset of labor, usually less than 12 hours prior to delivery [2]. However, most stillbirths are preventable with about half occurring in the intrapartum period. Studies have shown that antepartum stillbirths reflect quality of antenatal care, while intrapartum stillbirths reflect quality of delivery care [2–4]. Despite having enormous economic, social and health implications for parents and the society, stillbirths have been grossly under-reported and invisible in policies and programmes worldwide, with little recognition of potential strategies for intervention [3, 5].

Globally, the average stillbirth rate per 1000 total births declined from 24.7 in 2000 to 18.4 in 2015, equating to a 25.5% decrease in the number of stillbirths and an annual reduction rate (ARR) of 1.7% [6]. However, this reduction in stillbirths is slower compared to the progress in reducing maternal mortality (ARR = 3.0%) and under 5 mortality (ARR = 3.9%), for the same period [6]. An estimated 2.6 million stillbirths were reported in 2015, of which 98% occurred in low- and middle-income countries, 75% in sub-Saharan Africa and south Asia with 60% occurring in rural areas within these regions. Sub-Saharan Africa has reported the highest numbers of stillbirth and countries in the African Great Lakes region have contributed to this overall high numbers within the sub-continent [6, 7].

The African Great Lakes region which consist of Burundi, the Democratic Republic (DR) of the Congo, Kenya, Rwanda, Uganda and the United Republic of Tanzania is one of the world’s most densely populated areas. However, the region has been plagued by decades of political instability and armed conflicts, porous borders and humanitarian crises, as well as tensions over natural resources and other potentially destabilizing factors [8]. Such a deleterious environment has exposed inhabitants in the region to challenging public health problems which affects maternal and child health resulting in poor births outcomes. Reports obtained from national surveys conducted by each country in the Great Lakes region highlighted some socio-demographic factors associated with neonatal, post neonatal, infant, child, and under-5 mortality with no mention of stillbirth [9–13].

Previous studies have shown that the causes of stillbirth are closely linked to those of maternal and neonatal mortality such as; congenital anomalies, placental abruption, placenta previa, uterine rupture, asphyxia, operative delivery, prolonged or obstructed labor, hypertensive disorders, hemorrhage, anemia, extremes of neonatal birth weight, fetal growth restriction, prematurity, fetal asphyxia, malaria, untreated syphilis, pneumonia, sepsis and other maternal infections which are potentially modifiable through antenatal programmatic interventions [14–19].These studies also speculate that these potentially modifiable causes could be mitigated by the quality of maternity care spanning from conception to delivery. However, besides these maternal and obstetrics causes, the impact of sociocultural-related factors on stillbirth cannot be ignored especially in resource-limited regions like the African Great Lakes region. Hence, the aim of this paper was to ascertain the most significant potentially modifiable and sociocultural-related factors associated with stillbirth in the African Great Lakes region in order to drive region-specific interventions. This study used the most recent Demographic and Health Survey (DHS) of six countries in the region to conduct a pooled analysis of predisposing factors associated with stillbirth within the region. Results from this study will open up more discussions around preventable stillbirths and enable governments and non-governmental organizations to structure effective interventions targeted at improving maternal health from conception through to delivery.

## Methods

This study utilized datasets from the most recent DHS conducted in countries within the African Great Lakes region (Burundi, Congo DR, Kenya, Rwanda, Tanzania and Uganda). These datasets were pooled to ascertain the most significant factors associated with stillbirth across the Great Lakes region. However, DHS conducted in Congo DR did not collect data on stillbirth as a result Congo DR was excluded from the analysis. The DHS is a nationally representative survey which collect data on mortality, fertility, family planning and maternal and child health. The DHS programme uses standardized methods in their surveys to ensure uniformity of results from different countries. The survey employs a stratified, multi-stage (cluster), random sampling design. Information was obtained from eligible women aged 15–49 who participated in the survey in each country. Detailed survey methodology and sampling methods used in gathering the data have been reported elsewhere [9–13].

The analyses were restricted to stillbirths reported in the 5 years preceding the surveys in order to limit differential recall of events by the mothers. A birth history with reproductive calendar was used to estimate stillbirth in all the individual country DHS.

### Outcome measure

The main outcome measure for this study was stillbirth which refers to a baby born with no signs of life at or after 28 weeks' gestation [1]. The study outcome was reported as a binary variable with ‘Stillbirth’ coded as 1 and ‘Live birth’ coded as 0. Stillbirth was identified using reproductive calendar information such as duration of pregnancy and pregnancy outcomes.

### Study variables

**Fig 1:**
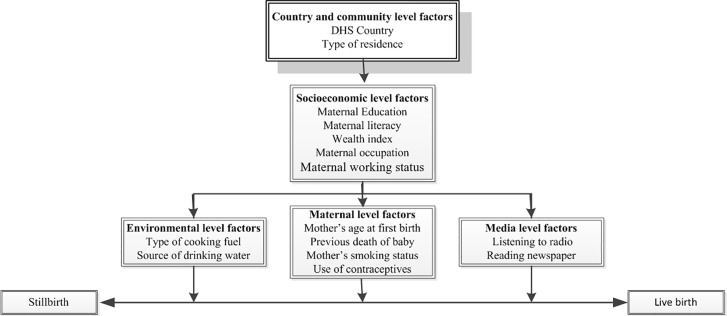
Conceptual framework for analysing factors associated with stillbirth in the African Great Lakes Region. Adapted from Mosley and Chen (1984).

The study variables used in this analysis were classified based on Mosley and Chen framework of factors influencing child survival in developing countries [20] into country and community level factors, socioeconomic level factors, maternal level factors, media level factors and environmental level factors as shown in Fig 1. Country and community level factors included the DHS country of survey and type of residence. The socioeconomic level factors were maternal education, maternal literacy, wealth index, maternal occupation, and maternal working status. The household wealth index serves as an indicator of the economic status of the household which is consistent with household assets, income and expenditure measures. The household assets and facilities used in calculating this index include: television (TV), refrigerator, bicycle, radio, motorcycle, car, electricity, type of toilet facility and type of building materials used in the place of dwelling. The index was represented as a score of household assets via the principle components analysis (PCA) method [21] and was categorized into five national-level wealth quintiles: poorest, poor, middle, rich and richest with the bottom 40% being referred to as the poorest and poor households, the next 20% as the middle-class households, and the top 40% as rich and richest households. Media level factors consist of listening to radio, reading newspaper and watching of TV. Maternal level factors were mother’s age at first birth, maternal body mass index (BMI), previous death of baby, maternal smoking status and use of contraceptives. Maternal BMI and watching of TV were excluded from the modelling because of large number of missing values which if included might bias the results. Environmental level factors were source of drinking water and type of cooking fuel. Source of drinking water was categorized into improved and unimproved sources according to WHO/UNICEF guidelines [22]. Improved water sources include piped water in a dwelling, plot or yard, public taps or standpipes, tube wells or boreholes, protected dug wells, protected springs and rainwater collection. Unimproved water sources include unprotected dug well, unprotected spring, cart with small tank/drum, tanker truck, bottled water and surface water (river, dam, lake, pond, stream, canal, and irrigation channels). Type of cooking fuel was classified as solid fuel and non-solid fuel. Solid fuels include (i) traditional biomass (wood, charcoal, agricultural residues, and dung), (ii) processed biomass (pellets, briquettes); and (iii) other solid fuels (such as coal and lignite). Nonsolid fuels include (i) liquid fuels (kerosene, ethanol, or other biofuels), (ii) gaseous fuels (natural gas, Liquefied petroleum gas, and biogas), and (iii) electricity [23].

### Data analysis

This study was based on secondary data analysis. The stillbirth rate and 95% confidence interval were estimated using the number of pregnancy losses during or after the seventh month of pregnancy for the 5 years preceding the interview, divided by the number of live births multiplied by 1,000.

Generalized linear latent and mixed models (GLLAMM) with the logit link and binomial family [24] that adjusted for cluster and survey weights were used to determine the factors significantly associated with stillbirth. A multivariable analysis was conducted using a five staged conceptual model as shown in Fig 1. Country and community level factors were first entered into the baseline model to assess their association with the study outcome and factors significantly associated with the study outcome were retained. In the second stage, socioeconomic level factors were added to the significant factors retained from the first model and factors significantly associated with the study outcome in the combined models were retained. This procedure was repeated for the third, fourth and fifth modelling stages with the inclusion of maternal level factors, media level factors and environmental level factors respectively. At each modelling stage, only factors with p-values <0.05 were retained. The adjusted risk of independent variables was determined by estimating the odds ratios with 95% confidence interval (Cl) and those with p < 0.05 were retained in the final model. In the GLLAMM analysis, the intra-cluster correlation (ICC) with standard errors (SE) were obtained for the final model. Collinearity was also tested and reported in the final model. To avoid any statistical bias, the results from the analysis were double checked by: (1) entering only potential risk factors with a p-value < 0.20 obtained in the univariable analysis for backward elimination process, and (2) testing the backward elimination method by including all potential risk factors.

### Ethics

This study was based on the analysis of existing datasets in the DHS repository that are freely available online with all identifier information removed. Hence, no ethics approval was required. However, the first author communicated with MEASURE DHS/ICF International, and permission was granted to download and use the datasets.

## Results

### Distribution of study variables

Table 1 shows the number of pregnancies of 7+ months’ duration by country and the distribution of the study variables in the African Great Lakes region. Kenya and Burundi reported 9,484 and 13,835 pregnancies respectively. Rwanda and Tanzania reported 8,127 and 10,163 pregnancies respectively, while Uganda reported 15,437 pregnancies. A total sample of 57046 pregnancies of 7+ months’ duration was reported across the region.

**Table 1:** Distribution of study variables (N = 57046).

Study Variables	N	%
***Country and community level factors***		
**Country of survey**		
Kenya	9484	17
Burundi	13835	24
Rwanda	8127	14
Tanzania	10163	18
Uganda	15437	27
**Type of residence**		
Urban	12061	21
Rural	44984	79
***Socioeconomic level factors***		
**Maternal education**		
Secondary or higher	11198	20
Primary	33158	58
No schooling	12690	22
**Maternal literacy** (N = 57037)		
Literate	38636	68
Illiterate	18381	32
**Wealth index**		
Rich	12215	21
Middle	23208	41
Poor	21623	38
**Maternal occupation** (N = 56987)		
Not working	8601	15
Skilled/Professional	13060	23
Agriculture	35326	62
**Maternal working status** (N = 57028)		
Currently not working	11744	21
Currently working	45284	79
***Maternal level factors***		
**Mother's age at first birth** (N = 56899)		
<20	29717	52
20–29	26279	46
30+	903	2
**Maternal BMI (N = 35499)		
≤ 18.5	1935	3
18–25	25486	45
25+	8078	14
**Previous death of baby**		
No previous death	42599	75
Had previous death	14446	25
**Maternal smoking status**		
Non-smoker	56568	99
Smoker	478	1
**Use of contraceptives**		
Currently using	22558	40
Currently not using	34487	60
***Media level factors***		
**Listening to radio** (N = 57028)		
^**†**^Yes	38951	68
^**†**^No	18076	32
**Reading newspaper** (N = 57028)		
^¶^Yes	11235	20
^¶^No	45793	80
**Watching of TV (N = 41592)		
^Δ^Yes	12348	22
^Δ^No	29244	51
***Environmental level factors***		
*Types of cooking fuel (N = 55675)		
Non-solid fuel	966	2
Solid fuel	54709	96
*Source of drinking water (N = 55675)		
Improved	39680	70
Unimproved	15994	28

*Percentages did not add up to 100% because of missing values.

**Large number of missing values, variable was excluded from modelling.

^†^YES = Listens to the radio at least once a week, ^†^NO = Does not listen to the radio at least once a week.

^¶^YES = Reads the newspaper, ^¶^NO = Does not read the newspaper.

^Δ^YES = Watches television at least once a week, ^Δ^NO = Does not watch television at least once a week

**Fig 2:**
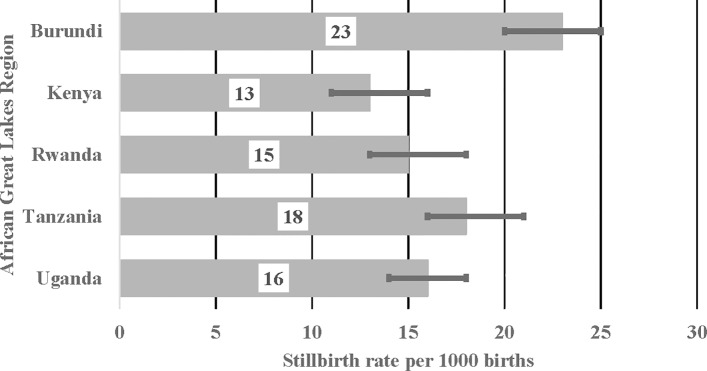
Stillbirth rates by country in the African Great Lakes Region.

Fig 2 shows stillbirth rate per 1000 births for countries in the African Great Lakes region. Burundi [23% (95% CI: 20, 25)] and Tanzania [18% (95% CI: 16, 21)] reported the highest stillbirth rate. However, the rate did not differ statistically because their confidence interval overlapped. Compared to Burundi, stillbirth rate was statistically lower in Kenya 13% [95% CI: 11, 16], Rwanda 15% [95% CI: 13, 18] and Uganda 16% [95% CI: 14, 18].

Table 2 shows the stillbirth rates and 95% Confidence Intervals (CI) by study variable within each country in the African Great Lakes region. Stillbirth was significantly higher in rural areas than urban areas in Burundi. Stillbirth was significantly higher among mothers with no schooling and mothers with secondary or higher education in Burundi, Rwanda, Tanzania and Uganda. Likewise, stillbirth was significantly higher among the poor households compared to the rich households in Burundi. Stillbirth was also significantly among mothers <20 years and 30+ in Burundi, Rwanda and Tanzania. Stillbirth was significantly higher among smokers across all 5 countries. Stillbirth was higher among mothers who did not use contraceptives in Kenya, Burundi, Tanzania and Uganda.

**Table 2:** Stillbirth rates and 95% CI in five countries in the African Great Lakes Region.

	Kenya (N = 9484)	Burundi (N = 13835)	Rwanda (N = 8127)	Tanzania (N = 10163)	Uganda (N = 15437)
Study variables	Rate(95%CI)	Rate(95%CI)	Rate(95%CI)	Rate(95%CI)	Rate(95%CI)
**Type of residence**					
Urban	14(12, 16)	16(14, 18)	16(14, 18)	16(14, 18)	14(12, 16)
Rural	13(11, 15)	23(21, 26)	15(14, 17)	20(17, 22)	17(15, 19)
**Maternal education**					
Secondary or higher	11(10, 13)	14(12, 16)	12(10, 13)	15(13, 16)	12(10, 14)
Primary	14(13, 16)	24(22, 27)	14(12, 16)	19(17, 21)	18(16, 20)
No schooling	13(12, 15)	23(21, 25)	24(22, 27)	20(18, 22)	20(18, 22)
**Maternal literacy**					
Literate	14(12, 16)	24(21, 26)	13(11, 15)	18(16, 20)	13(11, 15)
Illiterate	10(8, 11)	21(19, 23)	23(20, 25)	18(16, 21)	21(19, 24)
**Wealth index**					
Rich	14(12, 16)	14(12, 16)	15(13, 17)	17(15, 19)	14(12, 16)
Middle	14(12, 16)	26(24, 28)	11(10, 13)	21(19, 23)	19(17, 21)
Poor	12(10, 13)	21(19, 24)	20(18, 23)	18(16, 20)	15(13, 17)
**Maternal occupation**					
Not working	10(8, 11)	21(19, 24)	17(15, 19)	13(11, 15)	16(14, 18)
Skilled/Professional	11(10, 13)	15(13, 17)	15(14, 17)	23(20, 25)	16(14, 18)
Agriculture	17(15, 19)	24(21, 26)	15(13, 17)	18(16, 20)	17(15, 19)
**Maternal working status**					
Currently not working	11(9, 12)	21(19, 23)	10(9, 12)	13(12, 15)	18(16, 20)
Currently working	15(13, 17)	23(21, 25)	16(14, 18)	20(18, 22)	16(14, 18)
**Mother's age at first birth**					
<20	11(9, 12)	19(17, 21)	14(12, 15)	14(12, 16)	15(13, 17)
20–29	9(7, 10)	22(20, 24)	13(11, 15)	18(16, 20)	15(13, 17)
30+	9(7, 10)	36(33, 39)	39(36, 42)	23(21, 26)	10(8, 11)
Maternal BMI					
< = 18	8(6, 9)	32(29, 35)	31(29, 34)	7(6, 8)	13(12, 15)
18–25	13(11, 14)	21(18, 23)	13(11, 15)	18(16, 20)	13(11, 15)
25+	15(13, 17)	21(19, 23)	13(11, 14)	22(20, 24)	16(14, 18)
**Previous death of baby**					
No previous death	14(12, 16)	23(21, 25)	15(13, 17)	20(18, 23)	17(15, 19)
Had previous death	10(8, 11)	22(20, 24)	17(15, 19)	13(11, 14)	15(13, 17)
**Maternal smoking status**					
Non-smoker	13(12, 15)	22(20, 25)	15(13, 17)	18(16, 20)	16(14, 18)
Smoker	0(0, 0)	33(30, 35)	85(81, 90)	30(28, 33)	26(24, 29)
**Use of contraceptives**					
Currently using	9(8, 10)	19(17, 21)	14(12, 16)	14(12, 16)	13(12, 15)
Currently not using	18(16, 20)	24(22, 26)	17(15, 19)	21(19, 23)	18(16, 20)
**Listening to radio**					
Yes	14(12, 15)	23(21, 25)	16(14, 18)	19(17, 21)	17(15, 19)
No	12(10, 14)	22(20, 24)	13(11, 15)	16(14, 18)	14(12, 15)
**Reading newspaper**					
Yes	12(11, 14)	43(39, 46)	9(8, 11)	17(15, 19)	15(13, 16)
No	14(12, 15)	22(20, 24)	15(14, 17)	19(17, 21)	17(15, 19)
**Watching television**					
Yes	12(10, 13)	17(15, 19)	16(14, 18)	18(16, 20)	N/A
No	14(12, 16)	23(21, 25)	15(13, 17)	19(16, 21)	N/A
**Types of cooking fuel**					
Non-solid fuel	17(15, 19)	0(0, 0)	0(0, 0)	6(5, 7)	60(56, 64)
Solid fuel	13(11, 15)	22(20, 25)	16(14, 18)	19(17, 21)	16(14, 18)
**Source of drinking water**					
Improved	11(10, 13)	22(19, 24)	14(13, 16)	20(17, 22)	14(13, 16)
Unimproved	17(15, 19)	26(24, 29)	18(16, 20)	17(15, 19)	22(20, 25)

### Factors associated with stillbirth

In the univariate analysis, stillbirth was significantly associated with country of survey (Burundi and Tanzania), type of residence (rural), maternal education (primary and no schooling), maternal literacy (illiterate), wealth index (middle class), maternal occupation (agriculture), maternal working status (currently working), maternal age at first birth (30+), maternal smoking status (smokers), use of contraceptives and source of drinking water (unimproved).

In the multivariate analysis, Burundi was more susceptible to stillbirth compared with other countries in the region. Mothers with no schooling and with primary education are more likely to have stillbirth compared with mothers with secondary or higher education. Mothers who are above 30 years of age were more prone to stillbirth than those below 20 years. Mothers who smoke were more susceptible to stillbirth than non-smokers. Mothers who drank water from unimproved sources were more likely to have stillbirth than mothers who drank from improved water sources as shown in Table 3.

**Table 3:** Unadjusted and adjusted Odd Ratios (OR) for factors associated with stillbirths in the African Great Lakes region.

Study Variables	OR (95% CI)	P-Value	OR (95% CI)	P-Value
**Country of survey**				
Kenya	1.00		1.00	
Burundi	1.72 (1.32, 2.25)	<0.001	1.78 (1.38, 2.30)	<0.001
Rwanda	1.17 (0.87, 1.57)	0.312	1.23 (0.92, 1.65)	0.153
Tanzania	1.4 (1.06, 1.85)	0.019	1.43 (1.10, 1.88)	0.008
Uganda	1.23 (0.94, 1.62)	0.136	1.51 (1.17, 1.93)	0.001
**Type of residence**				
Urban	1.00			
Rural	1.25 (1.01, 1.54)	0.038		
**Maternal education**				
Secondary or higher	1.00		1.00	
Primary	1.46 (1.16, 1.83)	0.001	1.64 (1.32, 2.05)	<0.001
No Schooling	1.74 (1.36, 2.22)	<0.001	1.85 (1.44, 2.38)	<0.001
**Maternal literacy level**				
Literate	1.00			
Illiterate	1.21 (1.04, 1.41)	0.014		
**Wealth index**				
Rich	1.00			
Middle	1.31 (1.04, 1.64)	0.020		
Poor	1.21 (0.97, 1.52)	0.088		
**Maternal occupation**				
Not working	1.00			
Skilled/Professional	1.14 (0.88, 1.48)	0.330		
Agriculture	1.35 (1.07, 1.7)	0.012		
**Maternal working status**				
Currently not working	1.00			
Currently working	1.25 (1.03, 1.53)	0.026		
**Mother's age at first birth**				
<20	1.00		1.00	
20–29	1.10 (0.95, 1.29)	0.214	1.14 (0.99, 1.32)	0.077
30+	2.09 (1.36, 3.21)	0.001	2.39 (1.59, 3.59)	<0.001
**Previous death of baby**				
No previous death	1.00			
Had previous death	0.89 (0.75, 1.06)	0.190		
**Maternal smoking status**				
Non-smoker	1.00		1.00	
Smoker	2.16 (1.24, 3.75)	0.006	1.99 (1.19, 3.32)	0.009
**Use of contraceptives**				
Currently using	1.00			
Currently not using	1.52 (1.29, 1.78)	<0.001		
**Listening to radio**				
Yes	1.00			
No	0.97 (0.83, 1.14)	0.739		
**Reading newspaper**				
Yes	1.00			
No	1.09 (0.9, 1.32)	0.404		
**Types of cooking fuel**				
Non-solid fuel	1.00			
Solid fuel	1.01 (0.41, 2.44)	0.989		
**Source of drinking water**				
Improved	1.00		1.00	
Unimproved	1.19 (1.01, 1.39)	0.036	1.18 (1.01, 1.37)	0.035

In the final model, maternal education was replaced with wealth index and a significant association was reported between the poor [1.29 (95%CI: 1.04, 1.61)], the middle class [1.45 (95%CI: 1.18, 1.78)] and stillbirth. Maternal education was again replaced with type of residence and a significant association between rural residence [1.26 (95%CI: 1.03, 1.54)] and stillbirth was reported. Furthermore, non-use of contraceptives [1.17 (95%CI: 1.01, 1.35)] reported significant after replacing maternal education in the final model. In the final model, ICC = 0.27 and SE = 0.06. The estimated ICC of 27% is attributable to difference between cluster.

## Discussion

This study examined the rate of stillbirth across the African Great Lakes region to ascertain the intra-regional distribution of stillbirth in order to assist in the prioritization of interventions for countries with a higher burden of stillbirth. Burundi reported the highest rate of stillbirth in the region. The recent political instability in Burundi has resulted in a severely damaged health sector. Burundi's health system suffers from a lack of adequate medical infrastructure and human resources to meet urgent community health needs [25]. The high rate of stillbirth in Burundi could be addressed by targeting the quality of healthcare services provided by the health system during the antenatal period. However, the country’s government capacity to invest in the health sector is limited which grossly affects health service delivery. Therefore, to improve birth outcomes in Burundi, government and non-governmental organizations should focus on reinforcing the overall health system.

In this study, lack of maternal education was reported as a significant factor associated with stillbirth. Previous studies have shown that an increase in maternal education could lead to a corresponding improvement in healthy behaviors such as the timely decision to seek appropriate medical care during pregnancy [26, 27]. Studies have also shown that a higher maternal education could also increase health service utilization such as antenatal care services which involves serologic screening for syphilis, iron-folic acid supplementation, malaria treatment and prophylaxis, diagnoses and treatment of asymptomatic bacteriuria, blood pressure monitoring, anti-tetanus immunization, and the prevention of mother-to-child transmission of human immunodeficiency virus (HIV) thereby leading to a reduction in stillbirth [28–31]. Thus, this study provides supportive evidence that a lack of maternal education is associated with an increased susceptibility to stillbirth across the African Great Lakes region.

This present study also reported that advanced maternal age was significantly associated with stillbirth across the African Great Lakes region. This finding is consistent with results from other studies which show that advanced maternal age was associated with a range of pregnancy complications such as: placental abruption, fetal growth restriction, pre-term birth and preeclampsia which are known modifiable causes of stillbirth [32–34]. A systematic review conducted on the strength of association between advanced maternal age and stillbirth also reported a positive relationship between increasing maternal age and the magnitude of risk of stillbirth [35]. Contrary to the findings of this study, teenage mothers (>20 years) have been reported as being at a higher risk of stillbirth. Young mothers are prone to many health-related complications which arise not only from medical causes but have underlying sociocultural influences. These influences could result from lack of education, limited autonomy to make decisions due to family pressure and poor health-seeking behaviours [36–39]. Studies have reported a lower rate of early registration of pregnancy, use of health services, and frequency of routine antenatal check-ups and consumption of the recommended dosage of prophylactic iron-folic acid supplements among young pregnant women indicating that these mothers tend to be more negligent about their pregnancy either due to lack of awareness or immaturity [37–41]. However, a systematic review conducted on the predictors of stillbirth in low and middle income countries (LMICs) reported a high risk of stillbirths among mothers over 40 years of age which gradually decreased to its lowest point among mothers of 25–29 years old before increasing again among younger mothers [42]. Therefore interventions to reduce stillbirth should focus on both young and older mothers.

Maternal smoking during pregnancy has been well documented as being significantly associated with a range of obstetrics complications and poor pregnancy outcomes including stillbirth [43–46]. A systematic review of observational studies that measured the association between maternal smoking during pregnancy and the risk of stillbirth reported a dose-response effect of maternal smoking during pregnancy on risk of stillbirth indicating the more a mother smokes during pregnancy, the higher the risk of stillbirth [47]. Our finding adds to the body of research providing evidence that support the need to educate women on the risks of smoking to their unborn child.

Mothers who drank water from unimproved sources were more likely to have stillbirth. This may be as a result of environmental contamination of drinking water by chemical compounds which adversely affects birth outcomes [48]. It could also be due to suboptimal environmental sanitation which exposes underground water sources to bacterial contamination which on consumption could have adverse consequences on the foetus.

### Strengths and limitations

This study has a number of strengths. First, this study is population-based with a large sample size and a high response rate (> 90%) thus reducing a likely chance of selection bias. Second, the datasets used in this study are from nationally representative surveys and the variables are similarly defined in all surveys, hence comparable across all countries. Third, this study applied appropriate statistical adjustments to pooled data obtained from 5 nationally representative surveys and was able to identify the significant factors associated with stillbirth across the region to inform policies formulation.

Despite these merits, this study also has some limitations. First, due to the cross-sectional nature of the study design, this paper is unable to establish a causal relationship between stillbirth and the independent variables. Second, the diagnosis of stillbirth was from retrospective data based on self-report from mothers and this could be a source of recall and misclassification bias. Third, DHS do not collect data on modifiable risk factors associated with stillbirths which include maternal lifestyle such as maternal alcohol use or maternal obstetric complications such as antepartum haemorrhage, intra-uterine growth retardation, eclampsia, pre-eclampsia, congenital anomalies or maternal medical condition such as gestational diabetes, chronic hypertension, HIV, malaria, and genetic abnormality as well as maternal health service utilization such as folate supplementation and timing of commencement of antenatal care. Therefore, these factors could not be adjusted for during the analysis. A study by Tshibumbu and Blitz [18] examining modifiable antenatal risk factors for stillbirth amongst pregnant women in Namibia found that among a total of 14 modifiable risk factors included in the study, 11 (or 78.6%) were prevalent amongst women who had a stillbirth. It is possible that these factors also play an important role in explaining the burden of stillbirth in the Great Lake region; however, further studies are required to elucidate this. Finally, information on the limitations of the DHS data availability for stillbirth has been described elsewhere [49, 50].

Notwithstanding the above limitations, this study is useful and timely in identifying the factors associated with stillbirth across the African Great Lakes region to assist in proper public health planning. This is particularly important as the region is recovering from wars and our findings should inform development initiatives addressing maternal and child health to reduce socioeconomic inequalities in stillbirths. These findings will inform policy formulation around cost-effective socio-educational interventions aimed at improving foetal survival by increasing maternal education especially among women of advanced reproductive age in the African Great Lakes region.

## Conclusions

Stillbirth is a public health problem; however, it is highly preventable. This study shows the most significant factors associated with stillbirth across the African Great Lakes region were: maternal education (no schooling and primary education), advanced maternal age (30+ years), mother’s smoking status (smokers) and source of drinking water (unimproved). Therefore, community-based programmes promoting family planning initiatives and a healthy lifestyle are urgently needed to assist women in the proper timing of their pregnancies and to encourage healthy living especially among women with low education. This intervention will enable the region to achieve the Every Newborn Action Plan (ENAP) stillbirth target of 12 per 1000 births or less by 2030.

## Supporting information

S1 Table STROBE checklist. (DOCX)
